# Circulating proteins associated with allergy development in infants—an exploratory analysis

**DOI:** 10.1186/s12014-021-09318-w

**Published:** 2021-03-15

**Authors:** Marit Stockfelt, Mun-Gwan Hong, Bill Hesselmar, Ingegerd Adlerberth, Agnes E. Wold, Jochen M. Schwenk, Anna-Carin Lundell, Anna Rudin

**Affiliations:** 1 Institute of Medicine, Department of Rheumatology and Inflammation Research, Sahlgrenska Academy, University of Gothenburg, Box 480, 405 30 Göteborg, Sweden; 2 Institute of Clinical Sciences, Department of Pediatrics, Sahlgrenska Academy, University of Gothenburg, Gothenburg, Sweden; 3 Institute of Biomedicine, Department of Infectious Diseases, University of Gothenburg, Gothenburg, Sweden; 4 Affinity Proteomics, SciLifeLab, School of Engineering Sciences in Chemistry, Biotechnology and Health, KTH Royal Institute of Technology, Stockholm, Sweden

**Keywords:** Allergy, Children, Plasma proteomics, Longitudinal profiling, Risk factors, Prediction, Asthma, Atopic dermatitis, Allergic rhinitis, Food hypersensitivity

## Abstract

**Background:**

Protein profiles that can predict allergy development in children are lacking and the ideal sampling age is unknown. By applying an exploratory proteomics approach in the prospective FARMFLORA birth cohort, we sought to identify previously unknown circulating proteins in early life that associate to protection or risk for development of allergy up to 8 years of age.

**Methods:**

We analyzed plasma prepared from umbilical cord blood (n = 38) and blood collected at 1 month (n = 42), 4 months (n = 39), 18 months (n = 42), 36 months (n = 42) and 8 years (n = 44) of age. We profiled 230 proteins with a multiplexed assay and evaluated the global structure of the data with principal component analysis (PCA). Protein profiles informative to allergic disease at 18 months, 36 months and/or 8 years were evaluated using Lasso logistic regression and random forest.

**Results:**

Two clusters emerged in the PCA analysis that separated samples obtained at birth and at 1 month of age from samples obtained later. Differences between the clusters were mostly driven by abundant plasma proteins. For the prediction of allergy, both Lasso logistic regression and random forest were most informative with samples collected at 1 month of age. A Lasso model with 27 proteins together with farm environment differentiated children who remained healthy from those developing allergy. This protein panel was primarily composed of antigen-presenting MHC class I molecules, interleukins and chemokines.

**Conclusion:**

Sampled at one month of age, circulating proteins that reflect processes of the immune system may predict the development of allergic disease later in childhood.

**Supplementary Information:**

The online version contains supplementary material available at 10.1186/s12014-021-09318-w.

## Introduction

Allergy is the most common chronic disease among children in industrialized countries. At five years of age, about a third of Swedish and Danish children have been diagnosed with one or more allergic disease [1], including atopic dermatitis, asthma and allergic rhinoconjunctivitis. Early life events, including having elder siblings and growing up in a farming environment especially with dairy cattle are related to decreased risk of allergy development [2–4]. The hygiene hypothesis proposes that activation of the infantile immune system induces immune maturation that protects from subsequent allergy development [3]. In healthy individuals, the plasma proteome develops from birth to adulthood with significant changes in abundance and phosphorylation of several proteins [5]. Activation of the immune system during infancy could be expected to induce changes in plasma protein composition, however, the development of the plasma proteome in infants in the context of allergic disease has not been studied.

Parental history of atopy and sensitization predict the development of allergic disease in children [6, 7]. Further, our group has previously shown that high proportion of CD5 + B cells in infants predicts the development of allergic disease [8]. Multiple factors are likely to be involved, and there is no single biomarker in clinical use to predict the development of allergic disease. Proteomic approaches may be used prospectively to identify protein panels predictive of allergy development. Research in established allergic disease have revealed association between e.g. plasma chemokine ligand 5 (CCL5) and persistent asthma between 4 and 16 years of age [9] and upregulation of markers of inflammation such as myeloperoxidase (MPO), matrix metalloproteinase-9 (MMP9) and CCL22 in children with atopic dermatitis compared to healthy controls [10]. Apart from an association between lower plasma erythropoietin and soluble glycoprotein 130 at age 3 and asthma diagnosis at age 5 or 9 [11], comprehensive longitudinal protein profiling has not been used prospectively to predict allergy development in children. We hypothesized that further changes in the plasma proteome could be revealed by applying an affinity proteomics assay to screen for a large number of proteins [12]. Thus, an exploratory method was applied on consecutively collected plasma samples from infants in the FARMFLORA birth-cohort study [13] to assess circulating proteins for predicting the subsequent allergy development.

## Materials and methods

### Birth cohort study and collection of blood samples

We used samples from the prospective FARMFLORA study, a birth cohort of 65 full-term children followed longitudinally until 8 years of age, 28 who lived on dairy farms and 37 control infants from the same areas [13]. Of these children, 48 took part in the 8 year follow-up, and plasma samples were available from 44 children for this study. Demographic data are presented in Table 1. Heparinized blood plasma samples were available from umbilical cord blood (n = 38) and at 1 month (n = 42), 4 months (n = 39), 18 months (n = 42), 36 months (n = 42) and 8 years of age (n = 44). Parents provided written informed consent for their children and the study was approved by the Ethics Board of Gothenburg (Dnr 363–05 and 674–14).

**Table 1:** Demographic data of the study population

	N = 44
Girls, n (%)	23 (52)
Caesarean section, n (%)^a^	9 (20)
Birth weight in g, median (range)^b^	3543 (2715–4830)
Age of mother at delivery, median (range)	34 (21–42)
Sibling at birth, n (%)	22 (50)

^a^Four elective, three emergency, two unspecified caesarean sections

^b^Missing data from two participants

Parts of the data are published previously [13, 38, 39]

### Clinical examination

The allergy status was determined at 18, 36 months and 8 years by clinical examinations and parental interviews performed by study pediatricians as previously described [8, 13]. Allergic disease was defined as at least one of the following diagnoses: eczema, asthma, food allergy and allergic rhinoconjunctivitis cumulatively at 18, 36 months and/or 8 years of age as described in Additional file 2: Table S1. Clinical diagnosis of allergy in children at 18 months, 36 months and 8 years of age are shown in Table 2 and the children who were given a diagnosis of food allergy, eczema, allergic rhinoconjunctivitis and/or asthma are presented in in Additional file 3: Table S2. Of the 44 children, 18 children were diagnosed with an allergic disease at 18 months, 36 months and/or 8 years of age.

**Table 2:** Clinical diagnosis of allergic disease at 18 months, 36 months and 8 years of age

	18 months (n = 44)		36 months (n = 44)		8 years (n = 44)	
	*n*	%	*n*	%	*n*	%
Any allergy^a^	13	30	9	20	10	23
Food allergy	1	2	2	5	0	0
Eczema	11	25	6	14	5	11
ARC	1	2	1	2	3	7
Asthma	5	11	2	5	4	9

^a^One or more of the following diagnoses: food allergy, eczema, allergic rhinoconjunctivitis (ARC) or asthma

Parts of the data are published previously [8, 13]

### Exploratory protein profiling

The multiplexed affinity-based assay was performed at SciLifeLab (Stockholm, Sweden) with a suspension bead approach (SBA) using primarily polyclonal antibodies developed within the Human Protein Atlas [14] or purchased from commercial sources. Selected antibodies were coupled to magnetic color-coded microspheres (MagPlex, Luminex Corp, Austin, USA) and assessed for coupling efficiency, and an SBA was generated as previously described [15]. Beads coupled with anti-human albumin, donkey-anti human IgG or rabbit IgG served as positive controls, while un-coupled bare-beads served as negative control. Plasma samples were distributed by stratified randomization across 96-well plates and the analysts were blinded to patient variables including sex, age and allergy diagnosis. Each plate contained three replicates from two pooled samples and samples were labelled with activated NHS-PEO4 biotin, as previously described [16]. Labelled samples were diluted, heat-treated (56 °C, 30 min) and cooled, mixed with the SBA and incubated overnight whereafter R-phycoerythrin-labelled streptavidin (Invitrogen, Massachusetts, USA) was added. The beads were washed and analyzed on a FlexMAP3D instrument (Luminex Corp.). For each sample and bead identity, 50 events were counted. The protein binding was reported as median fluorescent intensity (MFI).

### SBA data processing and statistical analysis

For initial quality assessment, coefficient of variation (CV) was calculated from unprocessed data. Data were pre-processed for sample-by-sample variation with antibody-specific probabilistic quotient normalization (PQN) [17], while plate effects were adjusted using multi-MA normalization [18]. The normalized values from two repeated measurements were merged. The median coefficient of variation for each antibody between replicates across a 96-well plate or across a batch (384-plate) was ≤ 10%. Antibody-binders with mean fluorescent intensity (MFI) values below the rabbit IgG negative control bead or higher than the anti-albumin control beads were excluded. The data of one cord blood sample and two samples collected from children at 8 years of age could not be obtained properly and these were excluded. The MFI values were log-transformed, then scaled for each time point to have the same variance. To visualize the high-dimensional data, scaled Principal Component Analysis (PCA) using prcomp as function and t-distributed Stochastic Neighbour Embedding (t-SNE) with Rtsne (v.0.15) were performed. Two models, Lasso logistic regression and random forest, were tested using protein profiles at each time point, including sex and farming environment in the models. All statistical modelling was done on R (v.3.6.0). Logistic regression models were fitted to proteomic data with Lasso regularization using glmnet (v.3.0–2) package. Random forest models were built using randomForest (v.4.6–14). The models were trained and tested by 20-times repeated fivefold cross-validation with caret (v.6.0–86) R package.

## Results

### Age-related differences in abundant plasma proteins

**Fig. 1:**
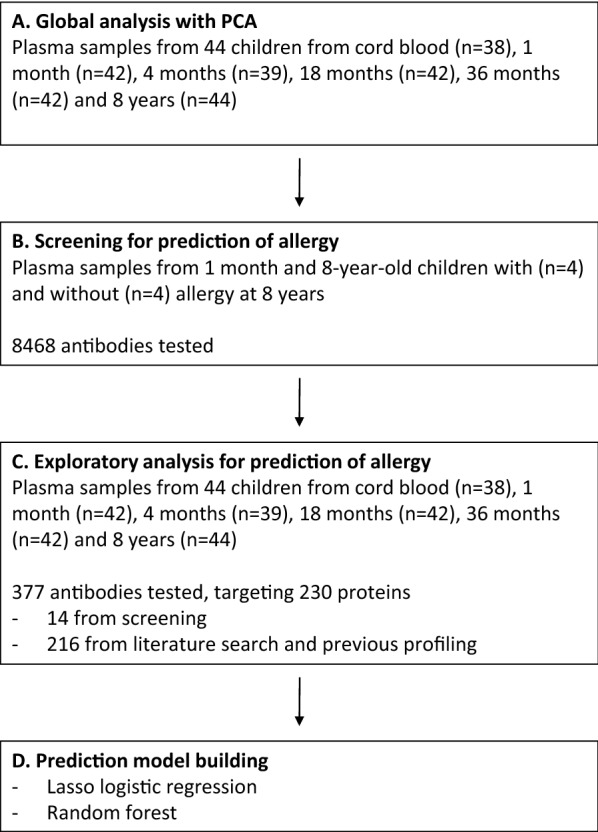
Study overview. **a** Global analysis with Principal component analysis (PCA) was performed with plasma prepared from umbilical cord blood and venous blood obtained at 1 month, 4 months, 18 months, 36 months and 8 years of age. **b** Screening of proteins in plasma was performed in eight children at 1 month and 8 years of age. **c** An exploratory analysis was performed on plasma samples obtained from cord blood and from venous blood collected from 1 month, 4 months, 18 months, 36 months and 8 year old children. **d** Prediction models for later development of allergy were evaluated using Lasso logistic regression and random forest

**Fig. 2:**
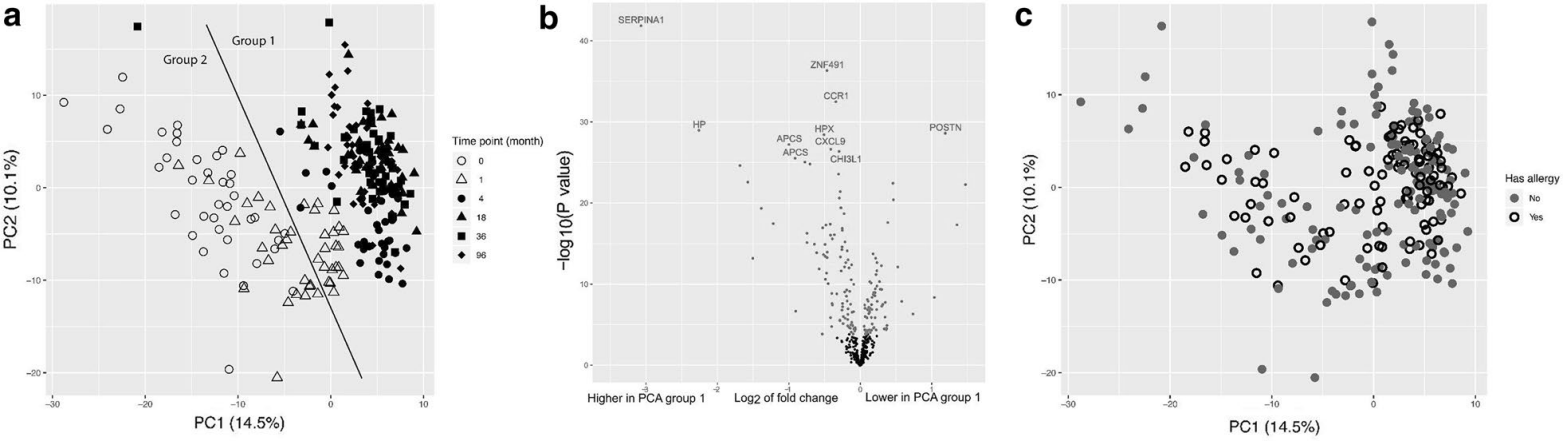
Age-related differences in plasma proteins. **a** Exploratory analysis with samples marked based on age, visualized with Principal Component Analysis (PCA) of proteins in plasma prepared from umbilical cord blood and venous blood collected at 1 month, 4 months, 18 months, 36 months and 8 years of age. **b** Volcano plot depicting protein associations with the two age-related clusters in the PCA analysis with the 10 most different proteins marked by name. Red colour represents significant associations with a Bonferroni corrected P-value < 0.05. **c** Exploratory analysis with samples marked based on allergy development or not, visualized with Principal Component Analysis (PCA) with plasma prepared from umbilical cord blood and venous blood collected at 1 month, 4 months, 18 months, 36 months and 8 years of age

A multistep procedure was used to identify proteins that could indicate protection or risk for the development of allergy (Fig. 1). To obtain an overview of the exploratory data from 44 children we conducted a PCA and found two main clusters that were linked to age at plasma sampling (Fig. 2a). All samples from cord blood and 24 out of 43 samples obtained at 1 month of age clustered together, while samples obtained at 4 months, 18 months, 36 months and 8 years of age clustered with little overlap to the samples obtained from cord blood and at 1 month. A tSNE analysis was performed with concordant results (Additional file 1: Figure S1).

Several of the obtained protein profiles (150 out of 370) differed significantly between the age-related clusters (false discovery rate < 0.01) (Fig. 2b). To investigate this further, the top 50 most different proteins were compared with those ranked at the bottom 50. Among the top 50, 28 proteins (56%) are secreted by the liver [19] including abundant proteins, defined as proteins with a concentration > 1 µg/ml, transport proteins and proteins of the complement system [20]. Among the bottom 50, however, only 3 proteins (6%) are secreted by the liver, while 10 proteins (20%) originate from blood cells and 20 proteins (40%) were cytokines/chemokines (Additional file 4: Table S3). When labelled according to allergy in the PCA analysis, samples from subsequently allergic and non-allergic infants appeared at random positions (Fig. 2c). In summary, abundant plasma proteins, often produced by the liver, differed by age, but were not associated with allergy development.

### Screening for protein profiles for prediction of allergy

To screen for circulating proteins that might predict allergy development, plasma samples from eight children, four of whom had developed allergy at 8 years of age were analyzed. Since epidemiological studies have shown that the first months of life are important for allergy development [2–4] and major immunological changes occur during the first three months of life [21], we selected samples from 1 month of age as the earliest postnatal time point to compare with 8 years of age as the latest time point of sampling. In order to screen for potential differences between allergic and non-allergic children, four children who had an allergic disease at all time points (i.e. 18 month, 36 months and 8 years of age) and four children who did not have any allergic disease at any of the time points were selected for the screening. The plasma samples were analyzed with bead assays using a total of 8468 antibodies [16], of which 8368 (99%) passed quality assessment. During the large scale screening, we identified 14 proteins based on a difference between allergic and non-allergic children of more than half/double median value. In addition, we conducted a literature search and selected another 216 proteins with relevance to inflammation and allergy. Altogether, 377 antibodies (Additional file 5: Table S4) targeting 230 proteins (Additional file 6: Table S5) were chosen to explore proteins associated with risk of or protection against allergy in a larger set of 44 children from the FARMFLORA cohort.

### Circulating proteins in samples collected at one month of age predict the development of allergy

**Fig. 3:**
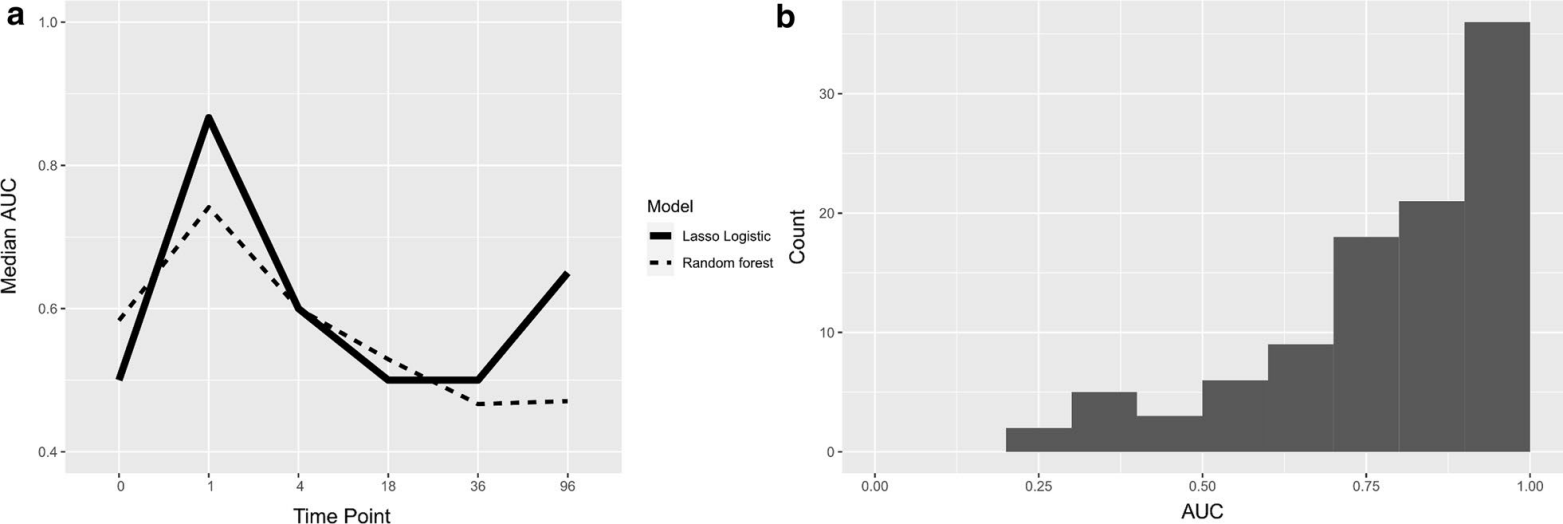
Circulating proteins at 1 month of age provided the best performing model for predicting allergy. **a** Median area under curve (AUC) for Lasso logistic regression and random forest prediction models for later development of allergy in samples obtained at birth (cord blood, n = 38) and at 1 (n = 42), 4 (n = 39), 18 (n = 42), 36 months (n = 42) and 8 years (n = 44). **b** Area under the curve (AUC) for the Lasso logistic regression model during 20-repeated fivefold cross-validation in samples obtained at 1 month

To determine at which time point the proteomic data would provide the best prediction model for the risk of later allergy development, the performance of two models, Lasso logistic regression and random forest analyses, were tested. Each model was fitted to randomly selected 4/5 of the protein profiles at each time point and tested with the remaining 1/5 of them, and the fivefold cross validation was repeated 20 times. The proteomic data from samples collected at 1 month of age resulted in the best performing models for prediction of allergy with median AUCs of 0.87 and 0.74 for Lasso and random forest models, respectively (Fig. 3a). The models fitted to the data from samples obtained at 4 months and 8 years of age predicted better than the random choice model, while the models fitted to the data from samples obtained from cord blood, 18 months and 36 months of age did not reveal any prediction capability with the AUC of 0.5. Most AUCs of the Lasso model at 1 month during cross-validation were close to 1, while only a few stochastic cases of AUCs close to and below 0.5 (8% AUCs < 0.5) were observed (Fig. 3b).

### A panel of 27 proteins measured at one month of age predict allergy development

**Fig. 4:**
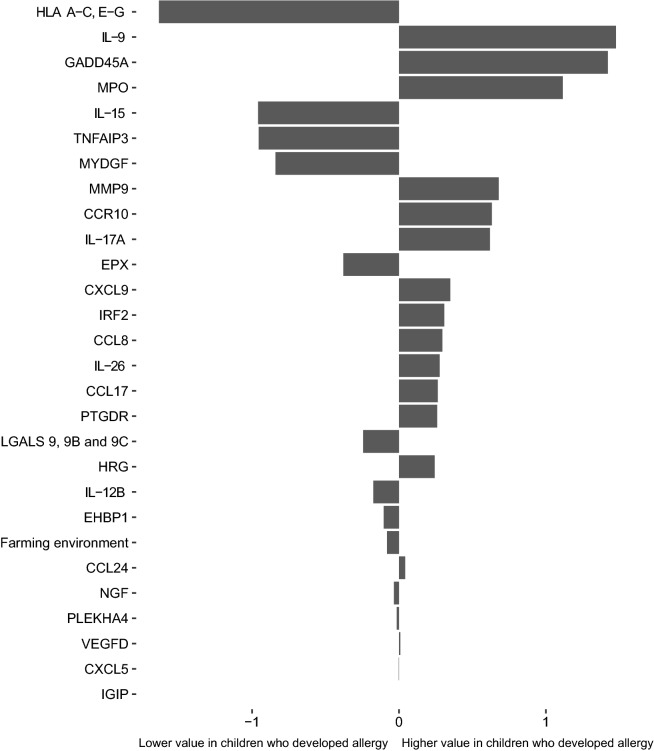
A panel of 27 proteins measured at 1 month of age predicted allergy development. The model that best differentiated children who later developed allergy or remained healthy was obtained in samples collected from children at 1 month of age. The proteins were sorted by the magnitude of the coefficients, which are the increments of log of odds ratios to be allergic per standard deviation elevation of the protein value. Positive coefficient indicates children with higher value of the profile had higher risk of developing allergy within 8 years

The Lasso model was tested using protein profiles at 1 month of age together with sex and farming environment. The Lasso model selected 27 proteins that together with farming environment best differentiated children who remained healthy and who later developed allergy (Fig. 4). The included factors were sorted by the magnitude of coefficients, which are the logarithmic increments in odds ratios to be allergic per 1 standard deviation increase in protein concentration. For example, the odds of developing allergic disease if the child had 1 standard deviation lower value of MHC class 1 were about 5 (= 1/*e*^−1.632^) times higher than the child with average value of the protein. The association of individual antibodies with allergic status was tested with t-test. All antibodies ranked by their statistical significance are listed in Additional file 7: Table S6.

The most prominent factor contributing to protection was found for MHC class I proteins, which were detected by an antibody targeting a common N-terminal epitope of the protein family. Likewise, children with higher value of Interleukin (IL)-15 as well as TNFα-induced protein 3 (TNFAIP3) and Myeloid Derived Growth Factor (MYDGF) at 1 month had lower risk to develop allergy during the 8 year follow-up period. Several interleukins constituted risk factors, including the largest contributing risk factor IL-9 as well as IL-17. Children with higher value of Growth Arrest and DNA Damage Inducible-α (GADD45A) as well as the proteases MPO and MMP9 also had higher risk to develop allergy. Finally, several chemokines and chemokine receptors were part of the profile, all with higher value in children who had higher risk for allergy development, with the strongest association found for chemokine receptor-10 (CCR10). These findings indicate that at the age of 1 month, serum levels of certain factors related to inflammation and immunological processes contain a predictive value regarding allergy development.

## Discussion

In this pilot study, we used exploratory affinity proteomics in a prospective birth cohort to search for proteins circulating in infant blood plasma that could indicate protection or risk for subsequent allergy development. We identified that samples collected at 1 month of age performed best in the prediction of subsequent allergy. Our findings highlight that early immune development, as reflected in the plasma proteome, may have importance for the later development of allergic disease.

Age at sampling had a strong influence on the plasma protein profile, which is in agreement with previous studies [22, 23], and half the samples from children at 1 month of age clustered with cord blood samples. Plasma proteins of high abundance, chiefly those produced in the liver, generated this difference in overall protein profile in relation to age. The multiplex assays that rely on labelling of the protein targets can be susceptible to changes in overall protein composition, whereby the degree of protein labelling may change due to the concentration of abundant proteins. Therefore, a probabilistic normalization approach was used that accounts for differences in global proteome composition as well as technical aspects of the analysis.

Few studies have explored the possibility to predict allergy development from the composition of the serum proteome [11], and the optimal time of sampling for the prediction of allergy is unknown. In the present study, samples collected at 1 month of age provided the best prediction model for subsequent allergic disease. This is in line with previous studies showing that early life exposures, e.g. contact with elder siblings or with farm animals in the first years of life strongly influence the risk of later allergic disease [2, 3]. The Lasso logistic model selected the factor farming environment to be included in the prediction model, but not sex. Although the prevalence of allergic disease is higher in boys compared to girls during early childhood, the differences between boys and girls are small [24–26]. In contrast, there is a clear allergy protective effect from farming environment [2, 4], which has previously been demonstrated also in this cohort [13]. Taken together, our results underline that the plasma proteome during the first month of life as well as allergy-protective environment is critical to study when creating prediction models for the development of allergic disease in children.

In samples obtained at 1 month of age, the Lasso model selected 27 proteins that best differentiated children who did or did not subsequently develop allergy. Proteins associated with the risk of allergy development were found in the low concentration range and included proteins with immune functions such as antigen presentation, cytokine signaling, chemotaxis and tissue remodeling. The largest protective effect was exerted by proteins of the MHC class I family. Soluble MHC molecules are increased in several inflammatory conditions including children with atopic dermatitis and may have immunomodulatory and antiviral properties [27]. The cytokine IL-15, which is produced by mononuclear cells and contribute to the protection against viral infection [28] was also a protective factor. This indicates that molecules involved in the early defence against viruses may beneficially affect an immune development that prevents allergy development. Further, two proteins involved in anti-inflammatory responses and tissue repair, TNFAIP3 [29] and MYDGF [30] respectively, were associated with lower risk of allergy development.

Risk factors for allergy development included IL-9 and IL-17A, cytokines which have been associated with asthma [31, 32]. The granule enzyme MPO, that is restricted to neutrophils and monocytes, also appeared to be predictive of allergy development. Further, MMP9, which is produced by inflammatory tissues and degrades extracellular matrix, promoting tissue remodeling, as well as GADD45A, a protein induced by DNA damage that promotes the transcription of MMP9 [33], were also present in increased amounts in children who later developed allergic disease. Serum MMP9 levels have previously been shown to be higher in adult asthmatic patients compared to controls [34] and both MPO and MMP9 are upregulated in children with atopic dermatitis [10]. Finally, several chemokines and chemokine receptors, most prominently CCR10, were upregulated in children who later developed allergy. The CCR10/CCL27 axis is important for the skin during homeostatic conditions, and circulating CCL27 is increased in children with atopic dermatitis [35]. Taken together, these results indicate that infantile changes related to allergic inflammation contain a predictive value for the development of allergic disease, and are in line with a very early immune dysregulation in children who later develop allergy [36].

There are also limitations to our study. One is the small study size, comprising only 44 children, which limits the statistical power and possibility to cover a wider range of heterogeneous molecular phenotypes. Further, we employ an affinity proteomics method that uses single binder analysis, hence we acknowledge the possibility of off-target antibody binding. The genetic contribution to the detection of the circulating proteome was also not assessed [37]. Validation experiments with other assays and in independent sample sets are necessary to confirm that the predictive model is firmly linked to allergy. The strength lies in the prospective nature of the study with sampling at several time points before allergy development and a thorough and repeated clinical examination for allergic disease.

In conclusion, we demonstrate a 27 protein profile in peripheral blood from children at 1 month of age that appeared promising for the prediction of subsequent development of allergy.

## Supplementary Information

**Additional file 1: Figure S1. **Age related differences in plasma proteins. Exploratory analysis with samples marked based on age, visualized with T-distributed Stochastic Neighbour Embedding (tSNE).

**Additional file 2: Table S1. **Diagnostic criteria for eczema, asthma, food allergy and allergic rhinoconjunctivitis.

**Additional file 3: Table S2. **Allergy diagnosis in allergic children at 18 months, 36 months and 8 years of age.

**Additional file 4: Table S3. **Proteins differing between samples from PCA clusters.

**Additional file 5: Table S4. **Antibodies used for exploratory analysis.

**Additional file 6: Table S5. **Proteome annotation of proteins in exploratory analysis.

**Additional file 7:** **Table S6. **Association to allergic status in samples collected at 1 month (T-test).

## Data Availability

The datasets used and/or analyzed during the current study are available from the corresponding author on reasonable request.

## Abbreviations

AUC: Area under the curve
CC: Chemokine ligand
CCR: Chemokine receptor
GADD45A: Growth Arrest and DNA Damage Inducible-α
IL: Interleukin
MHC: Major histocompatibility complex
MMP9: Matrix metalloproteinase-9
MPO: Myeloperoxidase
MYDGF: Myeloid Derived Growth Factor
PCA: Principal component analysis
SBA: Suspension bead approach
TNFAIP3: TNFα-induced protein 3
