# Supplementary figures and images for: Circulating proteins associated with allergy development in infants—an exploratory analysis
Source: Clin Proteomics. 2021 Mar 15;18:11. doi: 10.1186/s12014-021-09318-w (PMC7958444; doi:10.1186/s12014-021-09318-w)

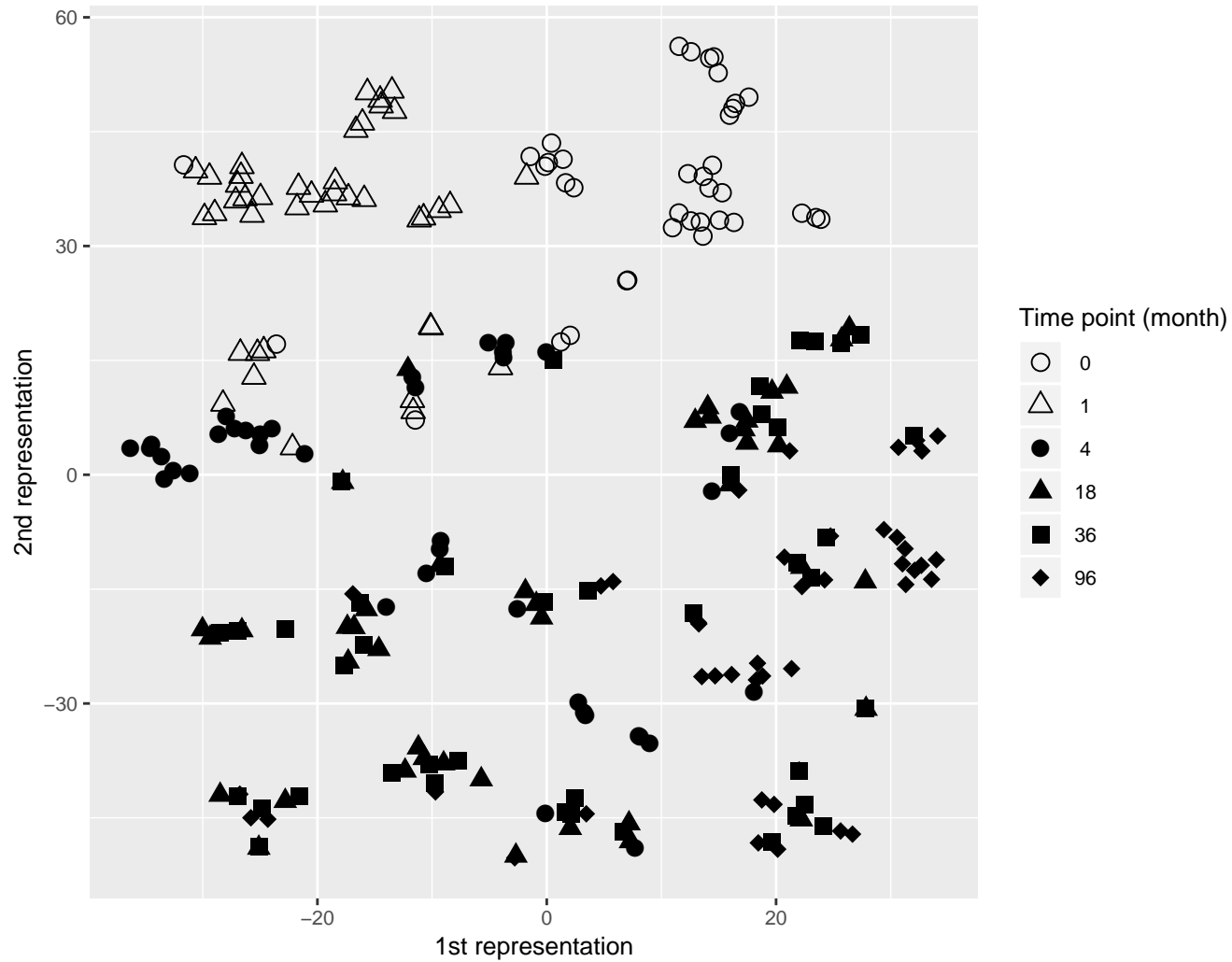

Supplement: Supplementary file 1 — Additional file 1: Figure S1. Age related differences in plasma proteins. Exploratory analysis with samples marked based on age, visualized with T-distributed Stochastic Neighbour Embedding (tSNE). [file 12014_2021_9318_MOESM1_ESM.pdf]
