# Supplementary material for: Circulating proteins associated with allergy development in infants—an exploratory analysis
Source: Clin Proteomics. 2021 Mar 15;18:11. doi: 10.1186/s12014-021-09318-w (PMC7958444; doi:10.1186/s12014-021-09318-w)
Supplement: Supplementary file 2 — Additional file 2: Table S1. Diagnostic criteria for eczema, asthma, food allergy and allergic rhinoconjunctivitis. [file 12014_2021_9318_MOESM2_ESM.docx]

**Additional file 3: Table S1**

|  | **Allergic rhinoconjunctivitis** | **Asthma** | **Eczema** | **Food allergy** |
| --- | --- | --- | --- | --- |
| **18 months** | Symptoms from eyes and/or nose upon exposure to pollen or animals together with a positive specific IgE or skin prick test to a corresponding allergen | ≥ 3 episodes of wheeze last year and any of:  -symptoms in between colds or  -other manifestations of allergy  Or persistent wheeze ≥ 4 weeks | Itching spots on typical locations which has come and gone for at least 6 months or  based upon William’s criteria | An immediate or late onset reaction after ingestion of the specific food, followed by a clear and prompt clinical improvement when eliminating the suspected food allergen, and any of:  -other signs of allergic disease or  -more than one organ system involved or  -supported by positive allergy tests, biopsies or challenge tests |
|  |  |  |  |  |
| **36 months** | Same as at 18 months of age, with symptoms occurring during the last 12 months | ≥ 3 episodes of wheeze with the last episode occurring after 2 years of age, and any of:  -symptoms in between colds or  -other manifestations of allergy  Or wheeze with onset after 2 years of age, and any of:  -triggered by colds together with other manifestations of allergy or  -triggered by exercise or  -response to anti-inflammatory maintenance therapy  Or persistent wheeze ≥ 4 weeks last year | Same as at 18 months of age, with symptoms occurring during the last 12 months | Same as at 18 months of age, with symptoms occurring during the last 12 months |
|  |  |  |  |  |
| **8 years** | Same as at 18 months of age, with symptoms occurring during the last 12 months | Wheeze/heavy breathing and any of:  -response to anti-inflammatory maintenance therapy or  -bronchial hyperresponsiveness on metacholine challenge (PD20 <0.6 mg) or  -bronchial obstruction reversible to β2-agonist ≥12% | Same as at 18 months of age, with symptoms occurring during the last 12 months | Symptoms of food allergy, supported by an open food challenge, planned or accidental |
|  |  |  |  |  |
