# Supplementary material for: Circulating proteins associated with allergy development in infants—an exploratory analysis
Source: Clin Proteomics. 2021 Mar 15;18:11. doi: 10.1186/s12014-021-09318-w (PMC7958444; doi:10.1186/s12014-021-09318-w)
Supplement: Supplementary file 3 — Additional file 3: Table S2. Allergy diagnosis in allergic children at 18 months, 36 months and 8 years of age. [file 12014_2021_9318_MOESM3_ESM.docx]

**Additional file Table S2**

|  | Age 18 months | | | | | Age 36 months | | | | | Age 8 years | | | | |
| --- | --- | --- | --- | --- | --- | --- | --- | --- | --- | --- | --- | --- | --- | --- | --- |
| Child ID | **FA** | **Eczema** | **ARC** | **Asthma** | **Sens** | **FA** | **Eczema** | **ARC** | **Asthma** | **Sens** | **FA** | **Eczema** | **ARC** | **Asthma** | **Sens** |
| 08 |  |  | x |  | x |  |  | x | x | x |  | x | x | x | x |
| 09 |  |  |  | x | x |  |  |  | x | x |  |  |  | x |  |
| 16 |  |  |  |  |  |  | x |  |  |  |  |  |  |  |  |
| 20 |  |  |  |  |  |  |  |  |  |  |  |  |  | x | x |
| 22 |  |  |  |  |  |  |  |  |  |  |  |  | x |  | x |
| 23 |  | x |  |  |  | x |  |  |  | x |  |  |  |  | x |
| 24 |  | x |  |  |  |  | x |  |  |  |  | x |  |  |  |
| 28 |  | x |  | x |  |  |  |  |  |  |  |  |  | x | ? |
| 34 |  | x |  | x |  |  |  |  |  |  |  |  |  |  |  |
| 35 | x | x |  |  | x | x | x |  |  | x |  |  |  |  | x |
| 36 |  |  |  |  | x |  |  |  |  | x |  | x |  |  |  |
| 45 |  |  |  |  | x |  | x |  |  | x |  |  | x |  | x |
| 46 |  | x |  |  |  |  | x |  |  |  |  | x |  |  |  |
| 49 |  | x |  | x |  |  | x |  |  |  |  |  |  |  |  |
| 50 |  | x |  |  |  |  |  |  |  |  |  | x |  |  |  |
| 58 |  | x |  |  |  |  |  |  |  |  |  |  |  |  |  |
| 60 |  | x |  | x |  |  |  |  |  |  |  |  |  |  |  |
| 64 |  | x |  |  |  |  |  |  |  | x |  |  |  |  | x |

Allergic rhinoconjuctivitis (ARC), food allergy (FA), sensitization (sens)

? test not performed

Parts of the data are published previously (Lundell et al, JACI 2015, suppl table)
